# Total body 100-mGy X-irradiation does not induce Alzheimer's disease-like pathogenesis or memory impairment in mice

**DOI:** 10.1093/jrr/rrt096

**Published:** 2013-08-01

**Authors:** Bing Wang, Kaoru Tanaka, Bin Ji, Maiko Ono, Yaqun Fang, Yasuharu Ninomiya, Kouichi Maruyama, Nakako Izumi-Nakajima, Nasrin Begum, Makoto Higuchi, Akira Fujimori, Yoshihiko Uehara, Tetsuo Nakajima, Tetsuya Suhara, Tetsuya Ono, Mitsuru Nenoi

**Affiliations:** 1 Research Center for Radiation Protection, National Institute of Radiological Sciences, 4-9-1, Anagawa, Inage-ku, Chiba 263-8555, Japan; 2 Molecular Imaging Center, National Institute of Radiological Sciences, 4-9-1, Anagawa, Inage-ku, Chiba 263-8555, Japan; 3 Research Center for Charged Particle Therapy, National Institute of Radiological Sciences, 4-9-1, Anagawa, Inage-ku, Chiba 263-8555, Japan; 4 Center for Nuclear Medicine and Ultrasound, Rajshahi H-18, Rajshahi Medical College Hospital Campus, Medical College Road, Rajshahi 6000, People's Republic of Bangladesh; 5 Graduate School of Medicine, Tohoku University, 2-1, Seiryo-machi, Aoba-ku, Sendai 980-8575, Japan; 6 Institute for Environmental Sciences, 1-7, Ienomae, Obuchi, Rokkasho-mura, Kamikita-gun, Aomori 039-3212, Japan

**Keywords:** total-body X-irradiation, low dose, Alzheimer's disease, Morris water maze test, Alzheimer's disease-like pathogenesis, mice

## Abstract

The cause and progression of Alzheimer's disease (AD) are poorly understood. Possible cognitive and behavioral consequences induced by low-dose radiation are important because humans are exposed to ionizing radiation from various sources. Early transcriptional response in murine brain to low-dose X-rays (100 mGy) has been reported, suggesting alterations of molecular networks and pathways associated with cognitive functions, advanced aging and AD. To investigate acute and late transcriptional, pathological and cognitive consequences of low-dose radiation, we applied an acute dose of 100-mGy total body irradiation (TBI) with X-rays to C57BL/6J Jms mice. We collected hippocampi and analyzed expression of 84 AD-related genes. Mouse learning ability and memory were assessed with the Morris water maze test. We performed *in vivo* PET scans with ^11^C-PIB, a radiolabeled ligand for amyloid imaging, to detect fibrillary amyloid beta peptide (Aβ) accumulation, and examined characteristic AD pathologies with immunohistochemical staining of amyloid precursor protein (APP), Aβ, tau and phosphorylated tau (p-tau). mRNA studies showed significant downregulation of only two of 84 AD-related genes, *Apbb1* and *Lrp1*, at 4 h after irradiation, and of only one gene, *Il1α*, at 1 year after irradiation. Spatial learning ability and memory were not significantly affected at 1 or 2 years after irradiation. No induction of amyloid fibrillogenesis or changes in APP, Aβ, tau, or p-tau expression was detected at 4 months or 2 years after irradiation. TBI induced early or late transcriptional alteration in only a few AD-related genes but did not significantly affect spatial learning, memory or AD-like pathological change in mice.

## INTRODUCTION

Alzheimer's disease (AD) is a progressive, irreversible neurodegenerative disease. As a common cause of dementia, it is characterized by formation of both senile plaques containing β-amyloid peptide (Aβ) derived from amyloid precursor protein (APP) and neurofibrillary tangles containing hyperphosphorylated tau protein (p-tau) in the brain that lead to progressive neuronal degeneration and death. AD is the leading cause of dementia among the elderly and the fourth leading cause of death [1]. In the USA alone, it is estimated that 4.5 million people have AD [2], and as many as 24 million people are affected worldwide [3]. The global prevalence of AD is predicted to double every 20 years to reach 80 million by 2040 [3]. Thus, it is absolutely crucial to better understand the various contributing factors and molecular pathogenesis of AD as part of an AD prevention strategy.

Despite the fact that AD was identified more than 100 years ago, its cause remains elusive. As with other common chronic diseases, AD probably develops as a result of multiple factors. Several risk factors for AD were identified in epidemiological studies in addition to age and female sex. The strongest and most consistent risk factor is the apolipoprotein E genotype epsilon 4 allele (ApoE4). Other risk factors include head injury, cardiovascular disease, low serum levels of folate and vitamin B12, raised plasma and total hormone levels, and family history. Occupational exposure to pesticides, solvents, and electromagnetic fields are widely studied, but the evidence for increased risk of AD is generally not consistent [4]. To date, although research has revealed a great deal about AD, with the exception of certain inherited forms of the disease, the causes of AD remain largely unknown.

There is increasing evidence, however, describing the effects of ionizing radiation (IR) on the brain, suggesting that exposure to IR is susceptible to ultimately favoring AD [5]. The brain is generally considered to be relatively resistant to IR because neurons are indeed resistant to radiation-induced cell killing [6]. However, a number of studies have described the existence of various physiological and cognitive effects of IR at various doses ranging from dozens of mGy to 61 Gy [6, 7]. Although radiation therapy is an important tool in the treatment of primary [8] and metastatic [9] brain tumors, it is also responsible for various adverse neurological effects such as cognitive dysfunction and dementia. Some reports have suggested that cognitive dysfunction, including dementia, can be observed in 20–50% of long-term brain tumor survivors older than 50 years old who were treated with radiotherapy [10–12]. Diagnostic medical exposure to radiation is the most rapidly increasing component of background radiation exposure to populations in developed countries due to the availability of computerized tomography scans in clinical practice [13]. Consequently, in addition to possible cancer induction, the issue of potential radiation-induced non-cancer diseases such as possible AD due to diagnostic medical exposures to low doses of low linear-energy-transfer (LET) IR is highly relevant [14]. Recent work of Lowe *et al.* suggested that even relatively low-dose exposures (such as the dose from computerized tomography scans) to mice could trigger mechanisms associated with cognitive dysfunctions seen in normal aging and AD in man [15]. Research on the human data from atomic bomb survivors has not reached a clear conclusion: although no apparent effect from exposure on cognitive function was found [16], the effect of increased risk of early death among atomic bomb survivors should be considered [17]. Therefore, it is of the utmost importance to study possible connections between exposure to IR and pathogenesis of AD [5] and to understand the biological effects of low-dose IR, which is emerging as a major neurological health concern.

The hippocampus is a major component of the brains of humans and other vertebrates. It belongs to the limbic system and plays important roles in the consolidation of information from short-term memory to long-term memory and also in spatial navigation. The hippocampus is a part of the cerebral cortex and contains two main interlocking parts: Ammon's horn and the dentate gyrus. While macroscopic changes in the hippocampus are easily observable after high-dose exposures to IR, low-dose irradiation could lead to cognitive dysfunction without significant morphological change [18]. Such cognitive changes are often manifested as deficits in hippocampal-dependent functions of learning, memory and spatial information processing [19–21]. As a brain area critical for learning and memory, the hippocampus is especially vulnerable to damage in the early stages of AD. Emerging clinical evidence has indicated that altered neurogenesis in the adult hippocampus represents an early critical event in the course of AD [5]. AD affects the hippocampus first and severely before affecting other parts of the cortex. Cognitive impairment, including disorientation, learning disability and memory loss, is usually the initial symptom in the early stage of AD. In addition to the association of alterations in neurogenesis with AD pathogenesis [22], from a functional point of view, the hippocampus also plays an important role in structural plasticity and network maintenance. Therefore, dysfunction resulting from early subtle disease manifestations may in turn exacerbate neuronal vulnerability to AD and contribute to memory impairment.

In the present study, possible AD-like alterations induced by 100-mGy X-rays were chronologically studied with a battery of examinations at transcriptional, behavioral and pathological levels in mice over a 2-year period after total body irradiation (TBI).

## MATERIALS AND METHODS

### Animals

Eight-week-old female mice of the C57BL/6J Jms strain were purchased from SLC, Inc. (Japan). The mice were maintained in a clean conventional animal facility under a 12-h light/12-h dark photoperiod (lights on 8:00 a.m.–8:00 p.m.). Animals were housed in autoclaved cages with sterilized wood chips and allowed free access to standard laboratory chow (MB-1, Funabashi Farm Co., Japan) and acidified water (pH = 3.0 ± 0.2) *ad libitum*. Animals were acclimatized to the laboratory conditions for two weeks as an adaptation period before use. To avoid possible effects related to the developmental condition of the animals, all 8-week-old mice with a significantly different body weight (more or less than the mean ± 2 SD) were omitted from this study. There were 70 mice each in the non-irradiated group and the irradiated group. In each group, the mice were randomly divided into three subgroups, namely, the subgroup for transcriptional study at 4 h and 1 year after TBI (12 mice), the subgroup for brain pathological study at 4 months and 2 years after TBI (12 mice) and the subgroup for behavioral study at 1 year and 2 years after TBI (46 mice).

As positive controls for detection of characteristic AD pathologies, two established AD mouse models were employed to ensure appropriateness of examination in the present study: transgenic Tg2576 mice (Taconic Farms, Inc. Hudson, NY, USA) overexpressing human APP with the Swedish mutation [23] and PS19 mice overexpressing human tau with the P301S mutation [24]. They were used as positive controls for detection of amyloid-related (APP and Aβ) and tau-related (tau and p-tau) pathologies, respectively. Female Tg2576 mice aged 22 months old were used for brain amyloid imaging with positron emission tomography (PET). Their brain tissues were used for positive immunohistochemical staining of APP and Aβ. Male PS19 mice aged 1 year old were used as the positive controls for immunohistochemical staining of tau and p-tau. Timing for collection of the brains from AD model mice was based on published studies [23–25].

All experimental protocols involving the mice were reviewed and approved (Experimental Animal Research Plans No. 07-1001-4 and No. 07-1049-15) by The Institutional Animal Care and Use Committee of the National Institute of Radiological Sciences (NIRS). The experiments were performed in strict accordance with the NIRS *Guidelines for the Care and Use of Laboratory Animals*.

### Irradiation

X-rays were generated with an X-ray machine (Pantak-320S, Shimadzu, Japan) operated at 200 kVp and 20 mA using a 0.50-mm Al + 0.50-mm Cu filter. An exposure rate meter (AE-1321M, Applied Engineering Inc., Japan) was used for the dosimetry. The dose rate for delivering the TBI was ∼160 mGy/min. The 10-week-old mice were held in acryl containers and were exposed to TBI at a dose of 100 mGy at room temperature.

### Antibodies

The primary antibodies employed in this study were mouse monoclonal antibodies against APP (clone 22C11, Chemicon Millipore, Billerica, MA, USA), mouse monoclonal antibody against Aβ (clone 6E10, Signet Laboratories, Emeryville, CA, USA), mouse monoclonal antibody against normal tau (T46, Invitrogen, Camarillo, CA, USA) and mouse monoclonal antibody against p-tau (clone AT8, Pierce Endogen, Rockford, IL, USA). The fluorophore-conjugated secondary antibodies were from either Molecular Probes (Eugene, OR, USA) or Life Technologies (Carlsbad, CA, USA).

### Polymerase chain reaction (PCR) array for mouse AD-related genes

The Mouse Alzheimer's Disease RT^2^ Profiler^TM^ PCR Array [26] was used in the present study. It profiles the expression of 84 genes important in the onset, development and progression of AD. The array includes real-time PCR primers for genes that contribute to Aß generation, clearance, and degradation, as well as genes involved in Aß signal transduction leading to neuronal toxicity and inflammation. In the present study, 5–6 mice in each experimental group were killed by cervical vertebral dislocation at 4 h or 1 year after TBI. The whole brains were removed from the cranial cavity, and then the hippocampi were collected and weighed. After washing with normal saline at 37°C, hippocampi were snap-frozen in liquid nitrogen (−196°C) for one week. Then the hippocampi were kept in dry ice and sent to Filgen, Inc. (Nagoya, Aichi Prefecture, Japan) where the PCR array was performed. In brief, the hippocampal tissues were homogenized using a homogenizer (Micro Smash MS-100; Tomy Seiko Co., Ltd, Japan), and total RNA was extracted with a Qiagen RNeasy Mini Kit (QIAGEN Sciences, Valencia, CA, USA). Total RNA quality check was performed with an Agilent 2100 Bioanalyzer using the Agilent RNA 6000 Nano Kit (Agilent Technologies, Santa Clara, CA, USA) and Agilent RNA 6000 Nano Ladder (Agilent Technologies) according to the manufacturer's protocols. The resulting RNA was reverse transcribed using the High Capacity RNA to cDNA Kit according to the manufacturer's protocol (Applied Biosystems, Foster City, CA, USA). PCR by use of TaqMan^®^ Array Mouse Alzheimer's Disease 96-well plates (Applied Biosystems) was performed on an 7900HT real-time PCR system (Applied Biosystems). Amplification reactions for each sample were performed in triplicate. The specificity of the PCR products was controlled by melting-curve analysis. The threshold cycle number (Ct value) for each gene obtained by real-time PCR was normalized to the Ct value of beta actin of the same sample, and the changes in expression for each gene were obtained with the delta-delta Ct method. For each gene, fold induction was calculated as the difference in gene expression levels between the irradiated and the non-irradiated group.

### Radiosynthesis and small animal PET imaging

N-^11^C-methyl-2-(4′-methylaminophenyl)-6-hydroxybenzothiazole (^11^C-PIB), which can react with amyloid fibrils and has a slow clearance from amyloid-rich regions when compared with that from normal regions, thus resulting in a high-level retention of radioactivity in amyloid-rich regions in brain, is used as a potential PET imaging probe for identifying AD amyloid pathology in the human antecedent to clinical onset of AD [27] and in laboratory AD model animals [28]. Protocols for radiosynthesis of ^11^C-PIB and performance of mouse brain PET imaging were described in our previous study [28]. The radiochemical purity and specific radioactivity of ^11^C-PIB at the end of synthesis exceeded 95% and 250 GBq/μmol, respectively. PET scans were performed with an animal scanner (microPET Focus 220, Siemens Medical Solutions, USA). In brief, mice were anesthetized with 1.5% (v/v) isoflurane, and a 30-G needle connected to a 1-ml polypropylene syringe via a length of polyethylene tubing was inserted into the tail vein. Emission scans were acquired for 60 min in a 3D list mode with an energy window of 350–750 keV, and ^11^C-PIB (27 ∼ 54 MBq) was immediately injected intravenously. Summation images for ^11^C-PIB from 30 to 60 min were reconstructed with maximum *a posteriori* reconstruction, and dynamic images were reconstructed with filtered back-projection using a 0.5-mm Hanning filter. Volumes of interest (VOIs) were placed on hippocampi and cerebella using PMOD^®^ image analysis software (PMOD Group, Zurich, Switzerland) with reference to the MRI template. Tracer uptake in each VOI was estimated as the percentage of injected dose per tissue volume (%ID/ml).

### Immunohistochemical examination

At 4 months or 2 years after TBI, some animals from both the 100-mGy X-irradiated group and the non-irradiated group were anesthetized by CO_2_ inhalation and then immediately killed by cervical dislocation. The whole brains were collected, formalin-fixed, paraffin-embedded, and cut into 10-μm coronal sections. The sections were deparaffined, dehydrated in phosphate buffered saline and then immunostained based on a standard protocol. All stained sections were examined and photographed using an All-in-One Florescence Microscope System (BZ-9000, Keyence, Japan).

### Morris water maze test

Spatial learning and memory (acquisition and retention of spatial memory) were assessed with a circular water maze video tracking system (Muromachi Kikai Co., Ltd, Japan). The water maze was a circular pool (120 cm in diameter and 30 cm in height) with a white featureless inner surface and was filled with water to a height of 26 cm. Water temperature (22 ± 1°C) remained constant throughout the experiments. The pool was divided into four quadrants of equal area. A white platform (9 cm in diameter and 25 cm in height) was centered in one of the four quadrants of the pool and submerged 1 cm below the water surface so that it was invisible at water level. Protocols for testing acquisition of spatial memory were adopted from previous studies [29, 30] with modifications. In brief, for the acquisition period, mice were given one trial per day over 15 consecutive days to determine the location of the hidden platform. A 100-s session was allowed for each learning trial. During each trial, the time taken by the animal (latency) to find the hidden platform was recorded with automated computer-attached image-tracking software. In addition, distance swum by the mouse and speed were also recorded. Parameters were averaged for each session of trials and for each mouse. Once the mouse located the platform, it was permitted to remain on it for 20 s. If the mouse did not locate the platform within 100 s, it was placed on the platform for 20 s and then removed from the pool. The point of entry of the mouse into the pool and the location of the platform for escape remained unchanged. The decrease or increase in latencies from day-to-day in trials represented long-term memory or reference memory. When the mouse located the platform within 100 s, the trial was designated a success. The success score (percentage of the number of mice succeeding to the total number of mice tested) in each group was used to evaluate the acquisition of spatial memory. The mice that received a continuous success score in the last two trials (14th–15th) in the acquisition of spatial memory test were used in the test for retention of spatial memory. This test followed 1 week later, and the animals were assessed once a week for 15 consecutive weeks with a 7-day interval between the two tests. Successful retention of spatial memory was designated as that when the mouse located the platform within 100 s. The success score (percentage of the number of mice succeeding to the total number of mice tested) in each group was used to evaluate the retention of spatial memory. To avoid possible effects from behavioral tests performed at Week 34 after TBI, different animals were used in the behavioral study at Week 86 after TBI.

### Statistical analysis

Statistical evaluation of the other data was carried out with the χ^2^ test and Student *t*-test, as appropriate. Statistical significance was assigned to a value of *P* < 0.05.

## RESULTS

### Effect of radiation on mouse physiological development

**Fig. 1.**
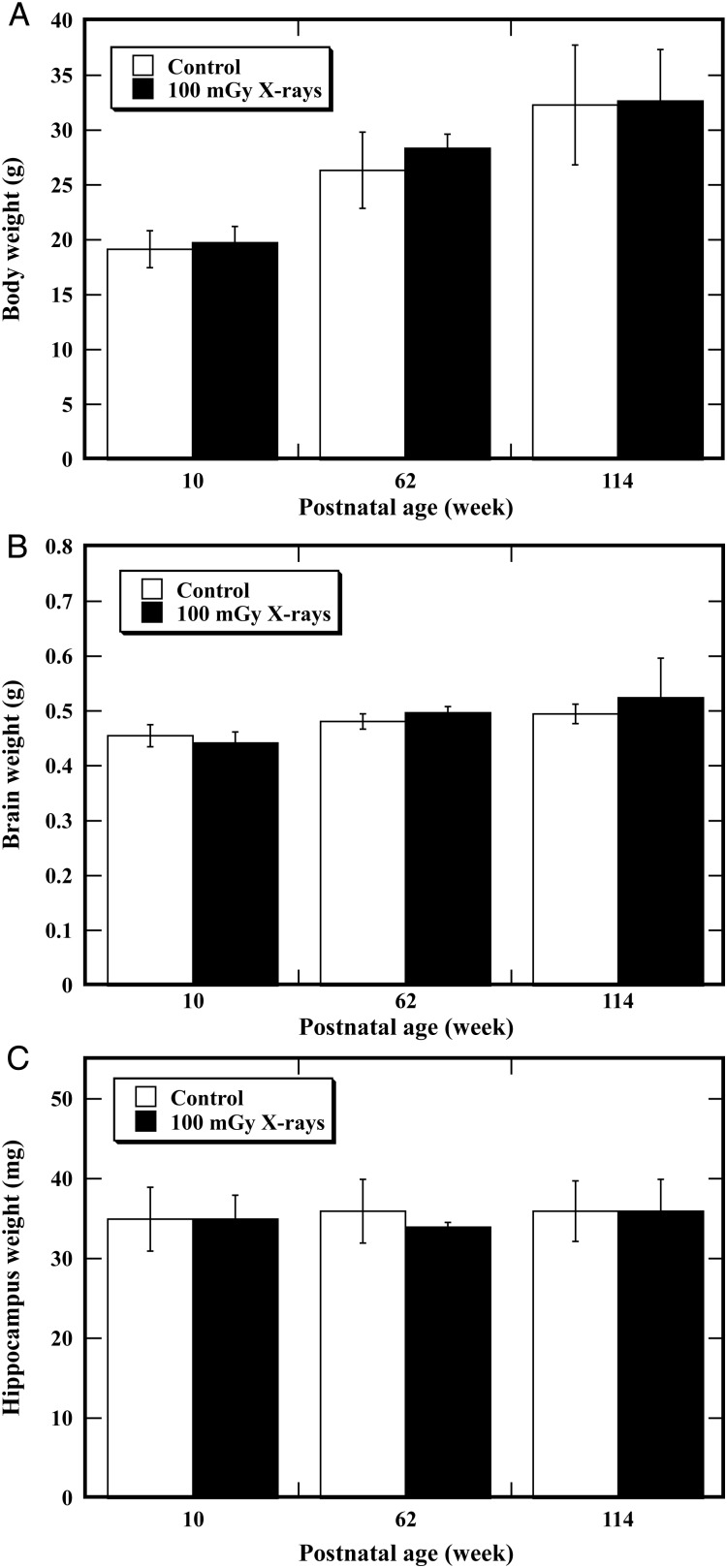
Mouse body weight gain, brain weight and hippocampus weight after total body irradiation (TBI). Mice were exposed to TBI at a dose of 100 mGy and their body weight (**A**), brain weight (**B**) and hippocampus weight (**C**) were measured, respectively, at 10, 62 and 114 weeks of postnatal age. Error bars were obtained from multiplication in the same experiment. For all these endpoints, no statistically significant differences were found between the unirradiated control and the irradiated animals.

To determine the possible effect of TBI on physiological development, body weight gain (Fig. 1A), brain weight (Fig. 1B) and hippocampus weight (Fig. 1C) were measured 4 h after irradiation, and at the postnatal ages of 62 and 114 weeks. No animal died of illness in either the unirradiated control group or the 100-mGy X-irradiated group. For all of the endpoints examined, no statistically significant differences were found between the unirradiated control and the irradiated animals at any of the time-points. These results indicated that TBI at a dose of 100 mGy could not induce any significant alteration in the physiological development of mice using body-weight gain, brain weight and hippocampus weight as the indices.

### Effect of radiation on expression of AD-related genes in mouse hippocampi

**Fig. 2.**
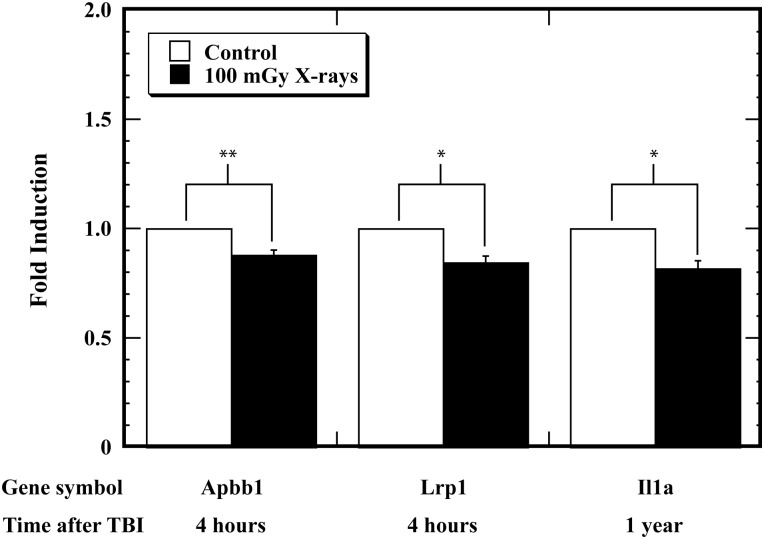
Transcriptional alteration in the expression of Alzheimer's disease (AD)-related genes in hippocampi in mice after total body irradiation (TBI). Mice were exposed to TBI at a dose of 100 mGy, and the transcriptional alteration in the expression of AD-related genes in hippocampi was studied at 4 h and one year after TBI. Error bars were obtained from multiplication in the same experiment. One asterisk (*) and two asterisks (**) indicate statistically significant differences at *P* < 0.05 and *P* < 0.01, respectively, compared with the unirradiated control.

To assess whether TBI is able to induce transcriptional alterations in AD-related genes, we measured the expression levels at 4 h and 1 year after irradiation of 84 AD-related genes that are involved in Aß signal transduction, generation, clearance and degradation in the hippocampus. Only two genes (*Apbb1* and *Lrp1*) at 4 h and one gene (*Il1α*) at 1 year after TBI were found to be significantly downregulated in the 100-mGy X-irradiated group compared with the unirradiated controls (Fig. 2). These results showed that TBI at a dose of 100 mGy could only alter transcriptional expression of a few AD-related genes in the mouse hippocampus.

### *In vivo* examination of amyloid fibrillogenesis with PET imaging in mouse brain

**Fig. 3.**
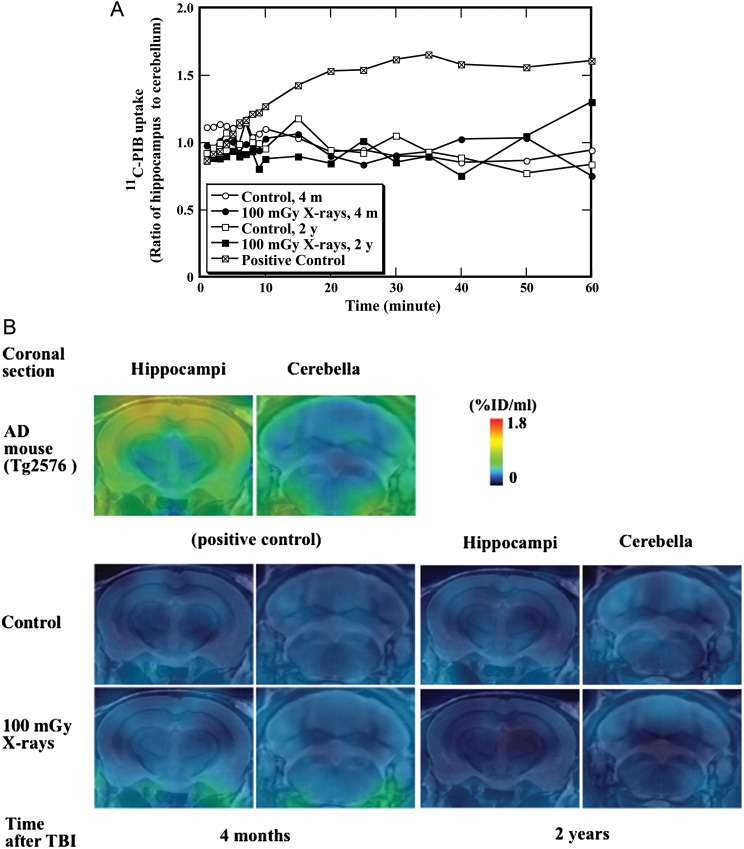
*In vivo* detection of fibrillar amyloid beta peptide (Aβ) in mice after total body irradiation (TBI). Mice were exposed to TBI at a dose of 100 mGy, and induction of fibrillar Aβ in mouse brain was studied at four months and two years after TBI. (**A**) The time-radioactivity curve of ^11^C-PIB is shown. (**B**) PET images were generated by averaging dynamic scan data at 30–60 min after administration of ^11^C-PIB and overlaid on the MRI template. Photos represent coronal images containing hippocamus and cerebellum regions at 2 and 6.5 mm posterior to the bregma, respectively. Images of postive control mouse (Tg2576) are shown in the upper panels.

Amyloid fibrillogenesis in mouse brain was examined with *in vivo* PET imaging, which is capable of providing longitudinal information of fibrillar amyloid deposition, at 4 months and 2 years after irradiation. Based on the hippocampus-to-cerebellum ratio of radioactivity at different time-points, ^11^C-PIB PET scan and summation PET images allow quantitative analysis of amyloid fibrillogenesis in the mouse brain. The representative time–radioactivity curves of ^11^C-PIB in the ratio of hippocampus to cerebellum are illustrated in Fig. 3A. Starting from 15 min after administration of ^11^C-PIB until the end of the measurement, the uptake of ^11^C-PIB in the positive control (female Tg2576 mice aged 22 months) was significantly higher than that in the animals from either the unirradiated control group or the 100-mGy X-irradiated group. The PET images were generated by averaging dynamic scan data (Fig. 3A) at 30–60 min after administration of ^11^C-PIB and overlaid on the MRI template (Fig. 3B). No significant difference in ^11^C-PIB uptake in the parietal cortex/hippocampus regions (normalized with cerebellum as reference tissue) was observed between the unirradiated control and X-irradiated animals at the ages of 4 months or 2 years, whereas a significant increase in ^11^C-PIB uptake was observed in the positive control (Fig. 3B). These data clearly indicated that no significant amyloid fibrillogenesis had occurred in the parietal cortex/hippocampus of the irradiated animals compared with the unirradiated control.

### Immunohistochemical analysis of amyloid and tau pathologies in mouse brain

**Fig. 4.**
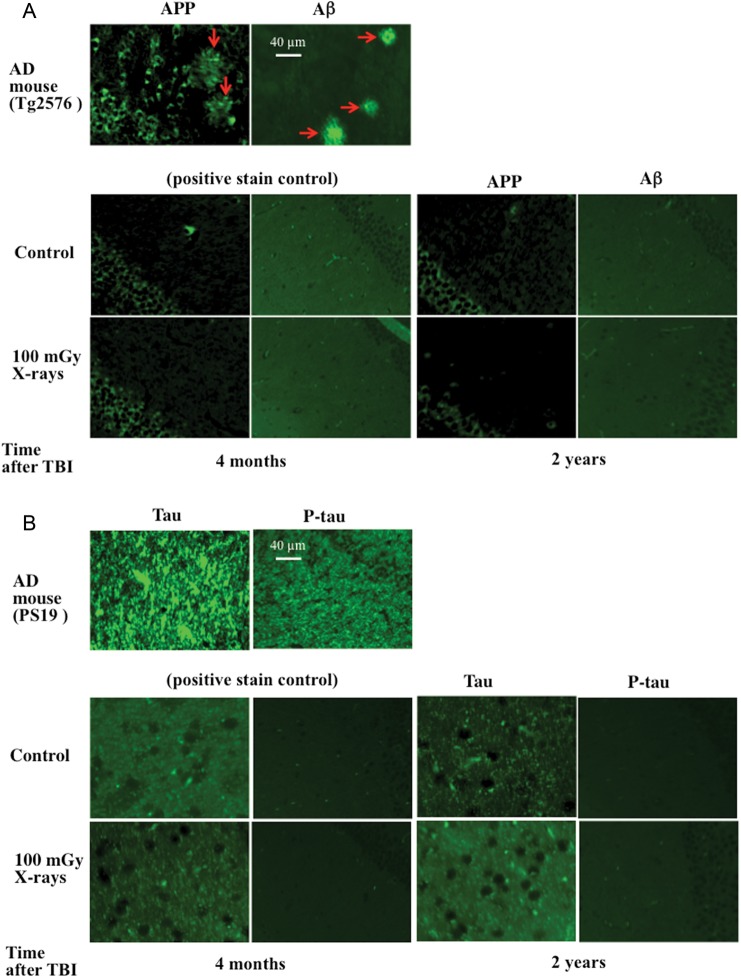
Immunohistochemical analysis for amyloid precursor protein (APP), amyloid beta peptide (Aβ), tau and p-tau in hippocampal CA1 regions of mice after total body irradiation (TBI). Mice were exposed to TBI at a dose of 100 mGy, and induction of APP and Aβ (**A**), and tau and p-tau (**B**) was studied at four months and two years after TBI. Overt immunoreactivities for APP and Aβ were detected in formed senile plaques (arrows) in the positive control mouse expressing amyloid-related pathologies (A, Tg2576 mouse). There was also great accumulation of tau and p-tau in the positive control expressing tau-related pathologies (B, PS19 mouse). No significant TBI-induced alterations in expression of APP, Aβ, tau and p-tau were detected in irradiated animals compared with the corresponding unirradiated controls.

Immunohistochemical analysis for APP, Aβ, tau and p-tau in the hippocampal CA1 regions of mice was performed *in vitro* at 4 months and 2 years after TBI due to its higher sensitivity for detecting weak to moderate pathological changes of AD compared with the *in vivo* PET assessment. In this study with laboratory mice, the immunohistochemical analysis was used to confirm the results obtained *in vivo* with PET examination. Mice were exposed to TBI at a dose of 100 mGy, and induction of APP and Aβ (Fig. 4A) and tau and p-tau (Fig. 4B) was studied at 4 months and 2 years after TBI. The CA1 area of the hippocampus is known to contribute to the acquisition of context-dependent extinction and is required for contextual memory retrieval [31]. It is one of the areas particularly vulnerable to degenerative processes in AD and displays AD pathologies in both AD patients and AD model mice [32, 33]. Results showed that in the hippocampus CA1 regions, APP and Aβ were positively stained only in AD model mice (Tg2576), and tau and p-tau were positively stained only in AD model mice (PS19). No overt accumulation of APP, Aβ, tau and p-tau expression was detectable in the irradiated animals compared with the corresponding unirradiated controls. These data confirmed the results obtained with PET and indicated clearly that no significant accumulation of APP, Aβ, tau and p-tau in hippocampal CA1 regions was induced by TBI in mice.

### Behavioral studies on acquisition and retention of spatial memory using Morris water maze test

**Fig. 5.**
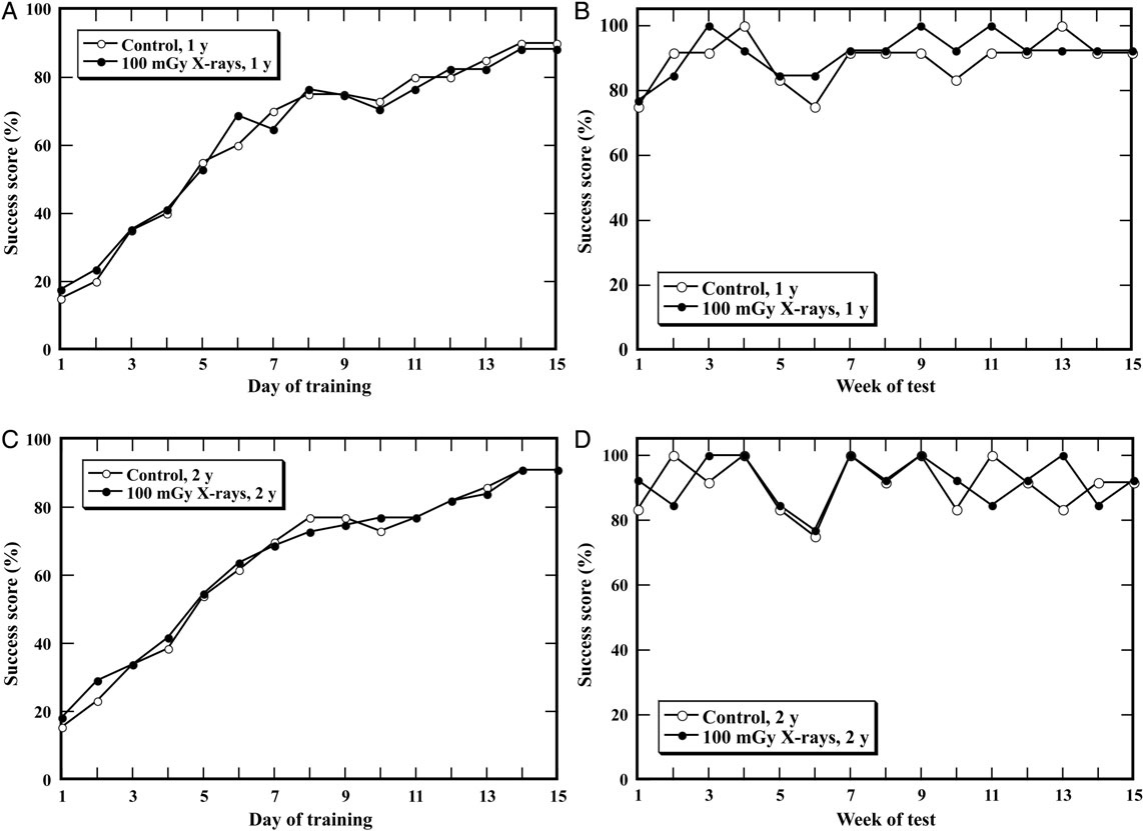
Test for acquisition and retention of spatial memory of mice after total body irradiation (TBI). Mice were exposed to TBI at a dose of 100 mGy, and a behavioral test with the Morris water maze was performed in the animals at postnatal ages of one year (**A, B**) and two years (**C, D**). No differences were found for either acquisition or retention of spatial memory in irradiated animals at these two time-points compared with those of the unirradiated control.

Mice were exposed to TBI at a dose of 100 mGy at the postnatal age of 10 weeks. To assess possible alterations in spatial learning and memory, behavioral testing with the Morris water maze was performed at the postnatal ages of one year (Fig. 5A and B) and 2 years (Fig. 5C and D) in the animals. No significant difference was found in the success scores for both acquisition and retention of memory in the irradiated mice compared with the control. To ensure that there was no difference between the control and the irradiated animals in regard to swimming or visual ability, the swim speed and the latency to reach the visible platform were also analyzed. No abnormalities were found in the irradiated animals for these two parameters when compared with the control (data not shown). These findings suggested that TBI at 100 mGy did not result in significant learning impairment or poor memory in either young or old mice.

## DISCUSSION

AD is a progressive, irreversible neurodegenerative disease with two well-characterized pathologies, senile plaques and neurofibrillary tangles. The main component of the senile plaques is Aβ resulting from the cleavage of APP. Ample evidence suggests that the accumulation of Aβ in the AD brain, resulting either from its increased production or reduced clearance, is associated with inflammation [34] and oxidative stress [35, 36] and ultimately results in AD-related cognitive impairment [37]. Although AD was first identified more than 100 years ago, research into its causes and risk factors has only gained momentum in the last 30 years. To date, only a small subset (<10%) of AD cases are known to result from an inherited autosomal dominant gene mutation; the cause and risk factors of AD are still largely unknown. Convincing evidence supports a role for oxidative stress in the pathogenesis of many chronic diseases [38]. Elevation of reactive oxygen species has been seen during AD pathogenesis, and exposure to IR could trigger oxidative damage [5]. Acute exposure to high doses of IR causes biological damage that results in severe health effects such as cancer. However, the effects and subsequent health implications of exposure to IR at low doses are largely unclear. Aside from diabetes [39], cardiovascular disease [40] and cancer [41], concerns raised also include late degenerative risk, including effects on the brain, such as AD. A previous study in mice reported early transcriptional response in brain to low-dose X-rays (100 mGy), suggesting alterations of molecular networks and pathways associated with cognitive functions, advanced aging and AD [15]. As humans are increasingly exposed to IR from various sources including medical diagnosis and outer space, the possible cognitive and behavioral consequences induced by low-dose IR are of great concern. The public health problem of AD is so great that an effort to evaluate any risk factor is probably justifiable. The present study investigated the acute transcriptional responses as well as the late pathological and behavioral consequences induced by IR at low doses in mice.

AD is a genetically complex neurodegenerative disorder associated with multiple genetic defects, either mutational or of susceptibility [42]. A number of studies have described gene modulations resulting from exposure to IR at various doses in a range of experimental models [43–48], including mouse brain [15, 49, 50]. A recent study has shown that exposure to low doses of IR triggers gene modulations that are different from those triggered by high doses, and involve genes associated with brain-specific functions, such as memory, learning and cognition [15]. Early IR-induced changes involve signal transduction mechanisms, ion regulation and synaptic signaling, whereas late changes involve metabolic functions, including myelin and protein synthesis [50]. Low-dose IR also modulates the expression of genes involved in stress response, cell-cycle control and DNA synthesis/repair [50]. In the present study, two genes, *Apbb1* and *Lrp1*, were found to be downregulated at 4 h after TBI, and one gene, *Il1*, was downregulated at 1 year after TBI. *Apbb1* is the gene symbol for amyloid beta A4 precursor protein-binding family B (Apbb) member 1. Proteins of *Apbb* family form multiprotein adaptor complexes with a range of functions. They all interact with the AD APP intracellular domain and can alter APP processing [51]. The protein encoded by the *Apbb1* is an adaptor protein localized in the nucleus. It interacts with APP, transcription factor CP2/LSF/LBP1 and the low-density lipoprotein receptor-related protein. Appb1 protein is brain-enriched. It is a multimodular adaptor binding to the cytoplasmic tail of the APP [52]. It modulates trafficking and processing of APP, including production of the Aβ. It also facilitates translocation of a carboxy-terminal fragment of APP to the nucleus and is required for APP-mediated transcription events. The Apbb1 protein could play an important role in the pathogenesis of AD [53]. In humans, a trinucleotide deletion in intron 13 of the *Apbb1* gene was associated with a slight risk for early-onset of AD [54], while in hippocampal area CA4, increased Appb1 protein immunoreactivity seemed to be associated with the severity [55]. In the brains of AD patients, expression of the mRNA isoforms of *Appb1* is broadly altered [56]. *Appb1* knockout mice have substantial neurodevelopmental defects showing impaired learning and memory [51, 57]. The members of the low-density lipoprotein receptor-related protein (Lrp) family have been implicated in the pathogenesis of AD. Lrp1, is also known as alpha-2-macroglobulin (A2M) receptor, ApoE receptor or cluster of differentiation 91. This protein localizes on the plasma membrane of cells, recognizes the complex of cholesterol and ApoE [58], and is involved in cross-membrane transportation of cholesterol. Importation of cholesterol into the neurons by ApoE via Lrp1 receptors is required to maintain the normality of neuronal functions. Impaired cholesterol importation damages neurons by starving them of cholesterol, which is thought to be a causal event in the onset of AD [59]. *Lrp1* is necessary for the A2M-mediated clearance of secreted APP and Aβ. Lrp1 protein is abundantly expressed in cerebrovasculature, in particular in vascular smooth muscle cells. It is a major Aβ clearance receptor in cerebral vascular smooth muscle cell, and a disturbance of this pathway contributes to Aβ accumulation [60]. Multiple susceptibility factors converge on metabolic pathways that involve Lrp1 protein. The uptake and degradation of both endogenous and exogenous Aβ were significantly reduced in Lrp1-suppressed human brain vascular smooth muscle cells. Conditional deletion of *Lrp1* gene in vascular smooth muscle cells in amyloid model APP/PS1 mice accelerated brain Aβ accumulation and exacerbated Aβ deposition as amyloid plaques and CAA, without affecting Aβ production. Elimination of Lrp1 expression in the CA fields and dentate gyrus of the hippocampus had no effect on the severity of amyloid deposition, the rate of Aβ40/42 accumulation, or the architecture of amyloid plaques in the hippocampus in APPswe/PS1dE9 transgenic mice, indicating that expression of Lrp1 protein by neurons in proximity to senile amyloid plaques does not appear to play a major role in modulating the formation of these proximal deposits or in the appearance of the associated neuritic pathology [61]. Simultaneous downregulation of *Lrp1* and *Apbb1* at 4 h after TBI is interesting because Lrp1 is known to interact with *Apbb1* [62]. The protein of the interleukin1alpha gene, or *Il1α*, is a member of the interleukin 1 cytokine family. It has been suggested that the polymorphism of this family of genes is associated with rheumatoid arthritis and AD. Il1α protein is a pleiotropic cytokine involved in various immune responses, inflammatory processes and hematopoiesis. It also functions as a cell-cycle regulator and a cell-signaling molecule and is involved in anti-apoptosis [26]. Il1α proteins are involved in the inflammatory response, and elevated levels of Il1α protein have been associated with a number of chronic diseases, including AD [63]. *Il1* is markedly overexpressed in the brains of patients with AD [64], although studies on AD patients have not shown the polymorphisms of the *Il1α* gene in a direct relation to AD [65]. As a pro-inflammatory cytokine, *Il1*α increased significantly in Aβ1-42- and Aβ25-35-treated rats [66]. Aβ induces Il1β mRNA expression, and Il1β is found in association with plaques in AD [67]. Although these three molecules have close association with the pathogenesis of AD, in the present study, transcriptional alteration in these genes appeared to be insufficient to lead to significant AD-like pathogenesis in the irradiated animals. The present result for gene expression was somewhat different from the previous work that showed significant changes in genes associated with memory, learning and cognition [15]. The difference may come from the different tissues assayed: in this study only the hippocampus was used while in that work the other brain tissues, e.g. the cerebral cortex and medulla were also included.

It is well known that exposure to IR at high doses (≥1 Gy) is detrimental to the organism. Studies using laboratory animals have shown that the underlying pathogenesis of the impairments involves loss of the neural precursor cells in the dentate gyrus of the hippocampus [53–55, 68–70]. However, effects from low doses have not been defined. Cumulative data suggest that a dose at 100 mGy can have an array of effects. High doses cause severe tissue destruction, and lower doses could induce cognitive impairments without signs of overt tissue damage. In the present study, TBI of mice with X-rays was performed at a low dose of 100 mGy. This dose was much lower than those mentioned in the reports above. The Morris water maze test has been a primary research tool to assess hippocampal-dependent spatial learning and memory in rodents for three decades. The task has proved to be particularly useful in demonstrating age-related memory impairment in transgenic mouse models of AD [71, 72]. We used success scores in the performance of the Morris water maze test to evaluate acquisition and retention of spatial memory as endpoints for detection of AD-like behavioral alterations in mice studied at the postnatal ages of approximately 1 and 2 years. No significant behavioral alterations were detected in the exposed mice at either age.

In the brain, overproduction and accumulation of Aβ are key pathogenetic events in AD progression, and presence of extracellular senile plaques is an important pathological feature of AD. Multiple lines of evidence demonstrate that overproduction and/or accumulation of Aβ aggregates in the brain is a primary cause of AD [73]. In the present study, amyloid imaging was performed, and no fibrillar amyloid was detectable in either young or old animals of the unirradiated control or the X-irradiated group during the observation period. These results were supported by the results of immunohistochemical analysis, in which no significant change in the expression of APP and Aβ was detected. An increase in the expression of normal tau and p-tau was also not detectable. These findings all supported the ultimate finding that there was no characteristic pathogenesis of AD in the brains of X-irradiated animals.

The opportunity is also increasing for humans to be exposed to high-LET irradiations due to research studies in outer space and radiotherapy, and it is known that high-LET irradiations induce different damage to the body in both quality and quantity, which may result in higher detrimental effects compared with that from low-LET irradiation. Exposure to high-LET irradiation is a potential health risk to the central nervous system in long-term space travel. The high-mass, highly charged and high-energy particles (such as Fe and carbon particles) are the most harmful component of galactic cosmic irradiation. The question of whether AD could be induced, or that its onset or prevalence might be influenced by high-LET IR, is of particular interest in the context of low-dose exposure of normal brain tissues during radiotherapy or activities in outer space. However, transgenic techniques have revolutionized our ability to develop animal models of AD and have also contributed significantly to the understanding of molecular pathogenesis [74]. Recent evidence indicates that low-dose low-LET irradiations could induce transcriptional alteration in AD-related genes in laboratory animals, and a low dose (100 mGy) of high-LET Fe irradiations could cause the early onset of AD in a mouse model of AD [75]. It should be noted that transgenic AD model mice were used, and these animals may respond differently to radiation exposure compared to wildtype animals. Another possible reason for the discrepancy between this work and our present study may come from the quality of the radiation. Further investigation on the possible effects on the brain from high-LET irradiation at low doses will be important for elucidating whether radiation of different qualities (such as LET value) is a risk factor for inducing AD.

## CONCLUSION

In summary, this study complements previously reported work examining the acute transcriptional response of mouse brain to 100-mGy X-irradiations by providing further insight into the late consequences using a battery of examinations at transcriptional, behavioral and pathological levels. A single dose of X-rays at 100 mGy could induce transcriptional alterations in a few genes, but it does not appear to cause any AD-like alterations in spatial learning and memory, or any AD pathogenesis in the brains of mice. These findings do not answer human health concerns about medical exposure to IR. It should be noted that the endpoints employed herein for transcriptional and behavioral studies are commonly only used in research work with laboratory animals; thus the extrapolation of these results to human health effects associated with exposure to IR requires further investigation.

## FUNDING

This work was supported in part by the Budget for Nuclear Research from the Ministry of Education, Culture, Sports, Science and Technology (MEXT) of Japan based on screening and counseling by the Atomic Energy Commission, and under contract with the Aomori Prefectural Government, Japan. N.B. was a fellow of the Nuclear Researchers Exchange Programme 2011, supported by the MEXT and the Nuclear Safety Research Association of Japan.
